# Sawdust for the Removal of Heavy Metals from Water: A Review

**DOI:** 10.3390/molecules26144318

**Published:** 2021-07-16

**Authors:** Elie Meez, Abbas Rahdar, George Z. Kyzas

**Affiliations:** 1 Department of Chemistry, International Hellenic University, 65404 Kavala, Greece; eliemouez@gmail.com; 2 Department of Physics, University of Zabol, Zabol 98613-35856, Iran; a.rahdar@uoz.ac.ir

**Keywords:** heavy metals, adsorption, sawdust, aqueous solution, thermodynamics, kinetics, isotherm, characteristics, parameters, cost

## Abstract

The threat of the accumulation of heavy metals in wastewater is increasing, due to their abilities to inflict damage to human health, especially in the past decade. The world’s environmental agencies are trying to issue several regulations that allow the management and control of random disposals of heavy metals. Scientific studies have heavily focused on finding suitable materials and techniques for the purification of wastewaters, but most solutions have been rejected due to cost-related issues. Several potential materials for this objective have been found and have been compared to determine the most suitable material for the purification process. Sawdust, among all the materials investigated, shows high potential and very promising results. Sawdust has been shown to have a good structure suitable for water purification processes. Parameters affecting the adsorption mechanism of heavy metals into sawdust have been studied and it has been shown that pH, contact time and several other parameters could play a major role in improving the adsorption process. The adsorption was found to follow the Langmuir or Freundlich isotherm and a pseudo second-order kinetic model, meaning that the type of adsorption was a chemisorption. Sawdust has major advantages to be considered and is one of the most promising materials to solve the wastewater problem.

## 1. Introduction

Since the twentieth century, the world has known a massive and rapid ascent in prosperity and technical advances, especially on the industrial scale; however, this fascinating evolution that the world has embraced has not been achieved without a big price that, unfortunately, the environment and all its elements has to pay. Many ecological problems threaten the health of the environment and the categories forming it, especially pollution. Pollution can be in different forms such as marine, soil, nuclear, thermal and even noise pollution. In recent years, extensive studies have been conducted to deal with a very serious problem, a problem that, if the world treats it carelessly, will have definite and major impacts on the environment. This threat is the accumulation of heavy metals in water, specifically wastewater and water sources. Most heavy metals are present normally in the environment and some of them are even believed to be essential to life, such as phosphorus, which can be found mainly in animal waste or in industrial effluents [1]. However, it is the presence of heavy metals as ions and in big concentrations in water that causes major concern due to their dangerous effect and toxicity on all land and aquatic life, including human life. This article will discuss heavy metals to understand their danger, their sources and some possible solutions.

Water is a very important element for the assurance of the existence of life and its continuity; it is very critical to protect fresh water since less than 1% of it can be sustained for human usage [2]. Heavy metals are used by some industries, such as textile industries, in their processes, and as catalysts and treatment chemicals [3]. The main sources of heavy metals are wide and include mining, chemical, tanneries, electroplating, semiconductors, batteries, metallurgical sources, electrical industries and even arts and crafts from developing countries [4,5]. Heavy metals are numerous and mainly include copper, zinc, lead, chromium, cadmium and nickel. Chromium, for example, can be found in steel production, cement, corrosive paints and dye [6]. It is very crucial to understand how toxic heavy metals are in high concentrations in order to preserve the continuity of living organisms, and already many regulations have been issued in order to prevent or at least minimize the concentration of heavy metals in waters and to control the effluents of the industries responsible for the generation of such elements.

Many studies show how dangerous heavy metals can be. For example, copper, which is a very important element in life, can also have toxic effects as it can contaminate water and foods such as chocolate, nuts and shellfish. An increase in the inhalation of copper can directly provoke lung cancer and large doses of copper can also cause irritation in the nervous system and in the liver and kidneys; moreover, copper is also highly linked with depression [7,8,9]. Another example is cadmium, which is not even essential and is highly toxic due to its ability to combine with sulfur and displace essential elements such as zinc and calcium [10]. Heavy metals not only pose a threat to human life but also contaminate the environment and are not biodegradable. This means that heavy metals will not transform into harmless elements with time and measures have to be taken to deal with their potential threat [11]. In China, increasing industrial and mining activities next to the Maba River have led to huge increases in the levels of heavy metals in the river which has worsened the quality of the Beijing River [12]. This situation puts lives of the inhabitants in this region in danger due to health concerns, since the quality of the drinking water has dropped and control measures of the discharge points of the effluents and their management should be taken [13]. It is also worth mentioning that one study showed that electroplating industries in China produce around 10 million tons of electroplating sludge on an annual basis [14]. Another example of the potential danger of heavy metals such as cadmium comes from studies which have shown that high concentrations of this heavy metal in rice led to the spreading of the Itaï-Itaï disease in 1955 [15,16] and the Minamata disease that spread due to fish contaminated with methylmercury [17], with both these diseases occurring in Japan.

Now that the capabilities of heavy metals to pose a threat to the environment have been covered and their sources of origin have been highlighted, the world is aware of this problem and its risks, which is why research has been conducted alongside various international implemented laws, regulations and legislations. Of course, the role of the majority of conducted studies is to find the best possible solutions to eliminate the threat of high concentrations of heavy metals or at least minimize it in order to preserve the environment. However, this is not enough, as regulations play an equally important role in order to control the disposal of effluents containing heavy metals and manage it, thus serving as a preemptive and necessary action to stop the threat from occurring in the first place. This is why some of the world’s top organizations that are tasked with protecting the environment and blocking any potential harm to its elements, such as the World Health Organization and the Environmental Protection Agency, cooperate with each other and issue acceptable quantities of heavy metals that could be discharged. This is very crucial and must be followed because any quantity of heavy metals disposed above the tolerated level could raise the toxicity of the disposed waste and cause severe damage to the environment and could be a major factor in health and mental problems [18]. Many regulations have been issued explaining the tolerable level of heavy metals, for instance a joint regulation between the Food and Agriculture Organization in the United Nations and the World Health Organization recommends a maximum acceptable level for intake of cadmium of 1.2 μg/kg of body mass [19], while lead ions and cadmium ions are restricted in drinking water to 0.01 mg/L and 0.02 mg/L, respectively [20]. A study in 2003 stated that in potable water, the recommended level of hexavalent chromium ions, also known as Cr(VI), is up to 0.05 mg/L [21]; furthermore, chromium in wastewater should be limited to 0.05 mg/L for hexavalent chromium and 5 mg/L to trivalent chromium, which is Cr(III) [6]. Naturally, such implementations are not totally applied worldwide and especially in new developing countries and third world countries, and this can be related to political systems that tolerate certain actions by tending to overlook the impact and importance of these regulations, or is due to the lack of knowledge in certain regions that allows the holders of the industries responsible for heavy metals discharge to ignore these regulations. However, it is an undeniable fact that such implementations, even though are not adopted entirely worldwide, have been able to regulate to a certain level the random discharge of the heavy metals in the environment, hence limiting the tremendous impact of such chaotic behavior on the environment and all its elements.

As mentioned before, the regulations stated by the top environmental organizations are very crucial for the continuity of life but, still, regulations alone cannot provide ultimate protection from the dangers of heavy metals accumulations and thus experiments and research must be conducted to find suitable solutions to eliminate high concentrations of heavy metals from potable water and wastewater alike. These scientific studies alongside the regularly provided regulations should be able to further diminish the threat of heavy metals accumulations and possibly eliminate it. There are many methods in order to remove heavy metals from wastewater and these methods have been subjected extensively to various studies in which different types of materials have been used in the objective of determining the best way to capture high accumulations of heavy metals. In the following sections, the various methods used for heavy metals collection will be discussed alongside different materials with a comparison between these materials. The main focus will be on the ability of sawdust to remove heavy metal ions from wastewater. Different aspects of sawdust, such as its structure, the ability to acquire heavy metals, efficiency, mechanism, parameters affecting the collection process and isotherms, shall be discussed.

## 2. Methods for Water Purification

In the previous section, it was briefly stated that scientists are constantly in an ongoing process to find methods that are suitable for the collection of heavy metals from waters. Mostly, these methods involve flotation, chemical precipitation, membrane separation, using minerals for adsorption, adsorption by activated carbon, electrolytic recovery, ion exchange, reverse osmosis, solvent extraction, cementation, electro deposition, ultrafiltration and electrochemical precipitation [6,22,23,24,25,26,27,28,29,30,31,32,33,34,35,36,37,38,39,40]. Although there are many techniques that can be used for the purification of water by removal of heavy metals, most of these techniques come with a certain number of difficulties that render them incapable of performing their required function. For example, chemical precipitation, which is one of the most used methods, is unable to remove concentrations of heavy metal ions that are low and, furthermore, it requires large settling tanks to deal with metal ions that are large in volume [41,42]. In fact, most of these methods cannot be implemented due to several factors including the high cost of operations, low selectivity of the metal ions, generation of sludge, the complicated nature of the treatment and their inability to achieve total water purification when concentrations of heavy metal ions are lower than 50 ppm [43]. In the literature, many methods have been reported in the past decades in order to remove heavy metal ions in the case of high accumulations but very few of these methods have been accepted and applied due to the many reasons mentioned above. This is why recent studies are focusing on more suitable and efficient methods that are able to give very good results when it comes to removal of heavy metal ions. The main direction is finding new methods and materials that are cost effective with bigger selectivity, hence leading to research on agricultural waste or agricultural by-products methods, which are mainly driven by the adsorption process in order to capture heavy metal ions from wastewaters.

## 3. Adsorbents for Heavy Metals Removal

### 3.1. Activated Carbon

Now that most of the studies conducted have proved to be inefficient due to various concerns, scientists have been reluctant about the adsorption process. Much research implies adsorbents to be the potential solution for removal of heavy metal ions from wastewaters and activated carbon has been a heavily studied and interesting topic. Mainly, there are four types of activated carbon, cloth activated carbon, granular activated carbon, fibrous activated carbon and powder activated carbon, with each one of them having its own characteristics and applications. Activated carbon has proved to be efficient for the removal of heavy metal ions from wastewaters since it has a large surface area and high surface reactivity, and one advantage of activated carbon is that it can be chemically modified by changing the surface area of the activated carbon; research has proved that chemically treated activated carbon has even higher removal percentage of heavy metals ions than unmodified activated carbon [22]. In fact, activated carbon has shown very good and promising results for the removal of heavy metal ions such as copper [44], zinc [45], cadmium [46] and nickel [47]. Other studies have also shown that activated carbon is an excellent extractor for hexavalent chromium, mercury and other metals [48,49]. However, when using activated carbon as an agent for the removal of heavy metals, although it is efficient, it can be very expensive, especially in developing countries due to high capital and operations costs [50].

### 3.2. Other Related Materials

Activated carbon is not the only adsorbent under investigation, as many studies have focused on other adsorbents that could be effective for the removal of heavy metals from wastewater. Some of the adsorbents that have been studied are zeolites, xanthate, fly ash, lignin, moss peat, chitosan, biomass, cactus material and clay [51,52,53,54,55,56,57,58,59,60,61,62]. All of the adsorbents mentioned above have big sorption capacity, but they are not suitable for wastewater treatment on the large scale as they are still expensive and their separation from wastewater is extremely difficult.

As it has been shown that different techniques lack efficiency and are not cost effective, and since activated carbon proved to be useful yet expensive regarding cost and separation measurements, it is notable to mention that much research has been extensively carried out, especially in the last 15 years, in order to find and identify new low-cost and at the same time efficient adsorbents for the removal of heavy metals from wastewater. These adsorbents include many materials that have been tested for cadmium ions removal from wastewater such as algae [63], coal fly ash [64], bone char [65], rice husk [66], yeast biomass [67], seaweed waste [68], sawdust [69,70], rice polish [71], spent grain [72] and brown marine microalgae [73]. Other investigated adsorbents have proved to be effective for certain heavy metal ions such mercury Hg(II), which is removed by flash ash [74], coal [70,75] and rice-husk ash [76]. Copper Cu(II) ions were found to be efficiently removed by maple sawdust [7] and soil and clay [77]. Other materials used as adsorbents to remove hexavalent chromium Cr(VI) are dried activated sludge [78], clinoptilolite [79], sphagnum moss peat [80] and fly ash wollastonite [81].

Most of these listed adsorbents were investigated by agricultural studies, which means that these materials are mostly agricultural waste or by-products. It is very important to pay attention to this category of sorbents and invest more resources in further improving our knowledge in this field because of the many advantages agricultural waste possess. The main problem of high heavy metal concentrations, besides their negative impact on human health and on the environment, is the fact that they are not biodegradable, hence making their disposal a significant issue. Fortunately, this is not the case with agricultural waste because this type of waste will degrade naturally into harmless elements, thus having no danger on the environment. However, agricultural waste poses a problem regarding disposal since it is difficult to get rid of this waste, especially when produced in large amounts. Therefore, it is also important to use agricultural wastes for adsorption of heavy metal ions since these wastes have a recognizable efficiency, are considered as largely unused resources and would further help future disposal problems.

## 4. Low-Cost Adsorbents

Since activated carbon has shown some cost-related problems, it was expected that the next studies would focus on low-cost sorbents to replace activated carbon and the other techniques that are used to recover heavy metals from wastewater. There are four categories of low-cost sorbents: agricultural waste, industrial by-products, natural materials and miscellaneous low-cost adsorbents [22]. All these materials are potential replacements for activated carbon in purification of wastewater and each category will be discussed briefly with comparison to the capabilities of activated carbon. Agricultural waste, such as orange peel, is widely available in nature and is very cheap since it has no economic value, which means that orange peel is not used in a known process and hence creates a disposal problem. In a study on removing nickel ions Ni(II) from simulated wastewater, it was shown that orange peel had a metal removal capacity of 158 mg/g [82]. Further chemical improvements can also be made to agricultural waste either by modifying the surface area or by heating it into activated carbon. The results suggest a major improvement in the metal uptake capacity of several agricultural wastes, thus rendering the chemical enhancement of agricultural waste a good potential method for heavy metals removal from wastewater. Of course, the cost of modifying agricultural waste is high but investigations show that the metal removal capacity obtained for these agricultural wastes may compensate the relatively high cost of chemical modification [83,84].

Industrial by-products can also be used for the removal of heavy metals from wastewater. These products share a lot of similarities with agricultural waste such as low cost and their abundance; the only difference is that they are not found naturally and can only be obtained by industrial processes. One study to assess the potential of industrial by-products was achieved by using green sands that can be collected from iron foundry industries. The study showed that green sands had a sorption capacity of zinc ions Zn(II) of 32.46 mg/g [85]. The results shown are lower than those of agricultural wastes but many studies have shown that other industrial by-products had similar adsorption capacities as the agricultural wastes adsorbents, which means the industrial by-products can compete with agricultural wastes for the removal of heavy metals from wastewaters [86].

Natural materials such as clays and zeolites, found in certain regions, can also be considered as low-cost sorbents and, due to their abilities to bind heavy metals, these materials have been investigated for water purification. Zeolites are used in Greece, for example, for the purification of water due to their low cost as it can be sold from 0.03 to 0.12 USD/kg depending on the quality of the zeolites [22]. Clays are classified into three types: bentonite, kaolinite and montmorillonite. Clays possess high cation exchange which means that they have an affinity to draw heavy metal ions; moreover, they are very cheap, as montmorillonite, for example, has a price ranging from 0.04 USD/kg to 0.12 USD/kg, which is twenty times cheaper than the price of activated carbon [22]. One study was conducted to investigate the clay adsorption capacity of copper, zinc and nickel ions and Table 2 shows the results [87]. The clay in this investigation was treated with HCL which improved the removal capacity of the mentioned ions. The results are good and the adsorption capacities are relatively high, with the highest adsorption capacity belonging to copper (83.3 mg/g) followed by nickel (80.9 mg/g) and zinc (63.2 mg/g). It can be concluded that clays can be considered as suitable low-cost adsorbents for the removal of heavy metals from water; however, the cost of such natural materials is higher than that of agricultural waste, besides the fact that agricultural waste still has a higher adsorption capacity which renders natural materials not suitable enough for commercial application as adsorbents. It is notable to also mention the miscellaneous low-cost adsorbents such as pyrolized coffee residue, which was investigated for removal of zinc, copper and cadmium ions from synthetic solutions [88]. The results of this experiment are presented in Table 1 and it is clear that coffee residue is a good adsorbent for the investigated ions, with copper ions being the most adsorbed (31.5 mg/g) followed by zinc (13.4 mg/g) and then nickel (11 mg/g).

Cochrane and co-workers [89] investigated the use of three biosorbents (crab carapace, macroalgae *Fucus vesiculosus*, peat) for the removal of copper from aqueous media. The results were directly compared with two commercial materials (activated carbon and ion-exchange resin). Langmuir and Freundlich isotherms were used to describe the adsorption equilibrium data. The Q_m_ values were 79.4, 114.9 and 71.4 mg/g for crab carapace, *F. vesiculosus* and ion-exchange resin, respectively.

**Table 1:** Low-cost adsorbents for heavy metal ions removal.

Adsorbent	Heavy Metal	Adsorption Capacity (mg/g)	Reference
HCl-treated clay	Zn(II)	63	[87]
	Cu(II)	83	[87]
	Ni(II)	81	[87]
Coffee residues	Zn(II)	13	[88]
	Cu(II)	31	[88]
	Ni(II)	11	[88]
Orange peels	Ni(II)	158	[82]
Green sands	Zn(II)	33	[85]
Clarified sludge	Cr(VI)	26	[90]
Rice husk	Cr(VI)	26	[90]
Activated alumina	Cr(VI)	25	[90]
Fly ash	Cr(VI)	24	[90]
Neem bark	Cr(VI)	20	[90]
Straw	Cr(III)	3	[40]
Olive stones	Cd(II)	49	[91]
Carrot residues	Cr(III)	45	[92]
	Cu(II)	33	[92]
	Zn(II)	30	[92]
Orange wastes	Cd(II)	48	[93]
Banana peels	Pb(II)	8	[94]
	Ni(II)	7	[94]
	Zn(II)	6	[94]
	Cu(II)	5	[94]
Orange peels	Pb(II)	8	[94]
	Ni(II)	6	[94]
	Zn(II)	5	[94]
	Cu(II)	4	[94]
Crab carapace	Cu(II)	79	[89]
Macroalgae Fucus vesiculosus	Cu(II)	115	[89]
Peat	Cu(II)	71	[89]

The removal of Cr(VI) from aqueous solution with a batch adsorption technique using different low-cost adsorbents was investigated by Bhattacharya et al. [90]. He used some low-cost adsorbents such as clarified sludge (a steel industry waste material), rice husk ash, activated alumina, fuller’s earth, fly ash, saw dust and neem bark to determine the adsorption efficiency for Cr(VI). The Langmuir model fitted the equilibrium data perfectly (R^2^~0.999) but demonstrated low adsorption capacities (19–31 mg/g): clarified sludge (26.31 mg/g), rice husk ash (25.64 mg/g), activated alumina (25.57 mg/g), fuller’s earth (23.58 mg/g), fly ash (23.86 mg/g), saw dust (20.70 mg/g) and neem bark (19.60 mg/g).

A strange adsorbent material (straw) was used by Kumar and co-workers [40] in order to remove heavy metals from aqueous systems. The straw was initially modified into alkali-treated straw (ATS) and insoluble straw xanthate (ISX), which slightly increased the cost of the adsorbent. The Q_m_ for the removal of Cr(III) was very low (1.88 and 3.91 mg/g for ATS and ISX, respectively).

Aziz and co-workers [91] investigated the adsorption of cadmium from treated olive stones (TOS) and after Langmuir modeling the calculated Q_m_ was 49.3 mg/g.

Heavy metals such as Cr(III), Cu(II) and Zn(II) were able to be removed from wastewater using HCl-treated carrot residues. Acid treatment was performed in order to remove tannins, resins, reducing sugars and colored materials. According to Nasernejad and co-workers [92], adsorption of metal ions into carrot residues was possible due to the presence of carboxylic and phenolic groups which have cation exchange properties. More metals were adsorbed at higher pH values of the solutions (pH 4 for Cr(III) and pH 5 for Cu(II) and Zn(II)). The maximum adsorption capacities were 45.09, 32.74 and 29.61 mg/g for Cr(III), Cu(II) and Zn(II), respectively.

Perez-Marin et al. showed that the untreated orange waste could only adsorb 48.33 mg/g Cd(II) [93]. Furthermore, adsorption of divalent heavy metal ions, particularly Cu^2+^, Zn^2+^, Co^2+^, Ni^2+^ and Pb^2+^, onto acid- and alkali-treated banana and orange peels was performed by Annadurai and co-workers [94]. The acid and alkali solutions used for modification of adsorbents were HNO_3_ and NaOH. In general, the adsorption capacity decreases in the order of Pb^2+^ > Ni^2+^ > Zn^2+^ > Cu^2+^ > Co^2+^ for both adsorbents. Banana peel exhibits higher maximum adsorption capacity for heavy metals compared to orange peel. The reported maximum adsorption capacities were 7.97 (Pb), 6.88 (Ni), 5.80 (Zn), 4.75 (Cu) and 2.55 mg/g (Co) using banana peel, and 7.75 (Pb), 6.01 (Ni), 5.25 (Zn), 3.65 (Cu) and 1.82 mg/g (Co) using orange peel. Acid-treated peels showed better adsorption capacities followed by alkali- and water-treated peels. Based on regeneration studies, it was reported that the peels could be used for two regenerations, for removal and recovery of heavy metal ions.

Comparing all the possible adsorbents with each other, it can be concluded that the most suitable low-cost adsorbents that can compete with activated carbon and truly contribute to high efficiency in heavy metal removal from wastewater are simple and chemically modified adsorbents from agricultural waste, since their heavy metals uptake are very similar to the adsorption capacity of activated carbon [22]. Although modifying agricultural waste adsorbents can be relatively costly, the results of this modification allow adsorbents from agricultural wastes to be economically viable since the price is still lower than activated carbon. Therefore, recent studies focused heavily on the abilities of low-cost adsorbents from agricultural waste for water purification from heavy metals and one of the main potential adsorbents that is being extensively under investigation is sawdust. In the following section, the structure, adsorption capacity, parameters affecting adsorption, mechanism, kinetics and thermodynamics of sawdust will be discussed.

All the above information is correct, but adsorption is a multi-parametric process, meaning that if one factor changes (i.e., pH, adsorbent dosage, temperature, etc.) then the final result will surely be different. So, any direct comparison among the adsorbents is not realistic, which consequently avoids any cost estimation.

## 5. Sawdust

### 5.1. Sawdust Characteristics

Sawdust is a very interesting material to investigate as an adsorbent for the removal of heavy metals from wastewater since it presents a lot of advantages. Sawdust is widely available as it is waste produced from sawmills and it is sold at a very cheap price. Furthermore, sawdust is biodegradable, which means that its disposal as waste should not endanger the environment or any of its elements. The structure of the sawdust alongside its components makes it a research subject regarding its adsorption process.

It is important to note that the behavior of sawdust depends on the chemical composition. The major chemical constituents of sawdust are hemicellulose, cellulose and lignin. On average, the quantitative percentages of hemicellulose, cellulose and lignin in sawdust are observed in the range of 15–35, 35–60, and 15–30%, respectively [95]. Table 2 summarizes the elemental compositions of various types of sawdust [95].

Table 3 shows a general approximation of the composition of sawdust. Sawdust contains mainly cellulose and lignin which are present in an acid detergent fiber that forms a part of the sawdust, and it also contains several hydroxyl groups, such as tannins. The importance of these elements mainly lies within their ability to attract cations by an ion exchange process [10].

Moreover, the particle size of a material influences many of its properties and can indicate the quality of the material and its performance. Whether for stability in suspension, reactivity, appearance, viscosity, flow, packing density, texture and flavor or many other characteristics, the particle size of a material is a very important component in understanding how a product performs. Particle size analysis can be as simple as measuring the diameter of a sphere or measuring the amount of material that will pass through a mesh of a specified size. It can also be a very complex analysis, examining the shape of the particles, its texture, and the distribution range of the various particle sizes. The information you need will determine which type of testing techniques to employ [96]. The effect of particle size is a complex phenomenon associated with significant change in physical and chemical properties of a substance due to direct reduction of the particles (grains, crystallites), the contribution of the interface to system properties and due to particle size being commensurate with the physical parameters of length dimension [96]. Size effects are observed with the reduction in size of structural elements, particles, crystallites and grain below a certain threshold. Such an effect occurs at the average size of crystal grain of less than 100 nm and is more clearly observed at the grain size below 10 nm. The specific surface area is increased as the particle size becomes small. The specific surface area is also increased if the particle has pores. The specific surface area is important for the industrial process and chemical reaction. Even with the same material that has the same weight and volume, the surface activity and adsorption volume are changed according to the specific surface area. So, it is important to measure the specific surface area to evaluate the activity and adsorption capacity of materials. (e.g., catalysis and adsorbent) [96].

**Figure 1:**
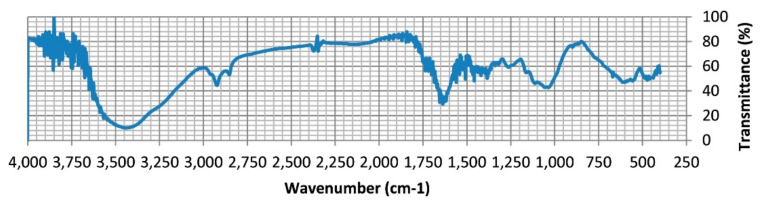
FTIR spectrum of pine sawdust. Reprinted with permission of Elsevier from Semerjian et al., 2018.

**Figure 2:**
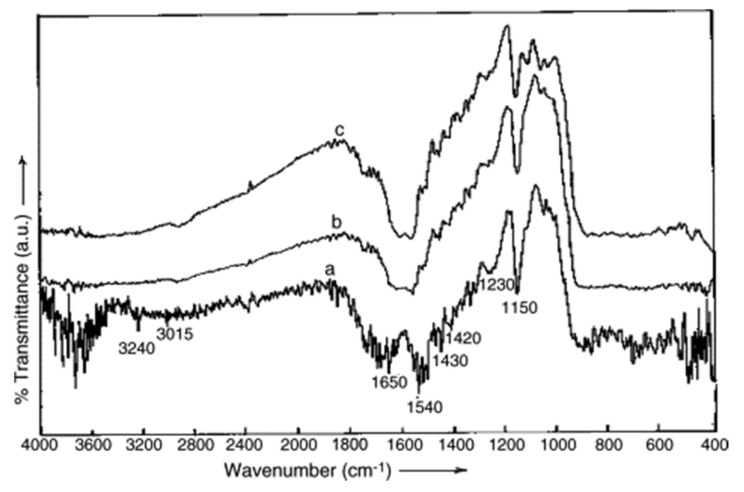
Fourier-transform infrared spectra of (**a**) untreated sawdust, (**b**) treated sawdust and (**c**) treated sawdust after adsorption. Reprinted with permission of Elsevier from Baral et al., 2006.

In order to determine these groups, several Fourier-transform infrared spectra have previously been conducted in order to view the different characteristics of the groups responsible for metal cations. An FTIR spectrum of pine sawdust (*Pinus halepensis*) is presented in Figure 1. The spectrum from Figure 1 shows several peaks ranging from 1030 cm^−1^ to 3430 cm^−1^; the big peak at 3430 cm^-1^ could define an overlapping between –OH and –NH stretching, and C–O and N–H vibration are detected hence proving the presence of hydroxyl groups and amine groups on the surface of the sawdust [97]. Figure 2 shows another Fourier-transform infrared spectrum belonging to another sample of sawdust. According to this, spectrum peaks were observed at 1160, 1250, 1420, 1450, 1540, 1650, 3015 and 3240 cm^−1^, which are attributed to OH stretching, OH deformation, CH deformation, amide, unsaturated groups such as alkenes, C–H groups and OH groups, respectively [98]. Both Fourier-transform infrared spectra prove that hydroxyl groups are an indispensable component of sawdust, thus emphasizing the importance of sawdust in attracting heavy metal ions.

**Figure 3:**
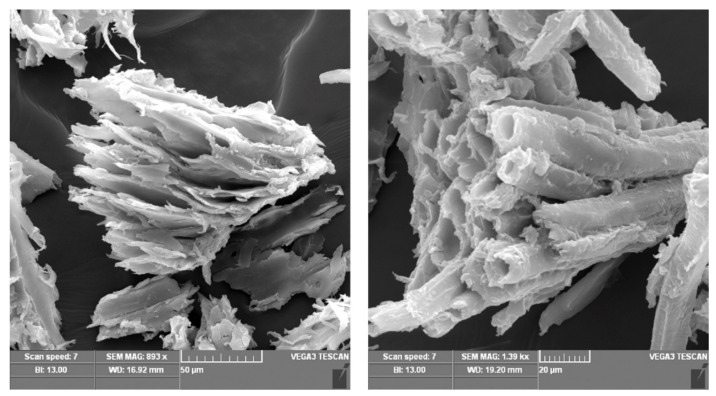
Scanning electron micrographs of *Pinus halepensis*. Reprinted with permission of Elsevier from Semerjian et al., 2018.

**Figure 4:**
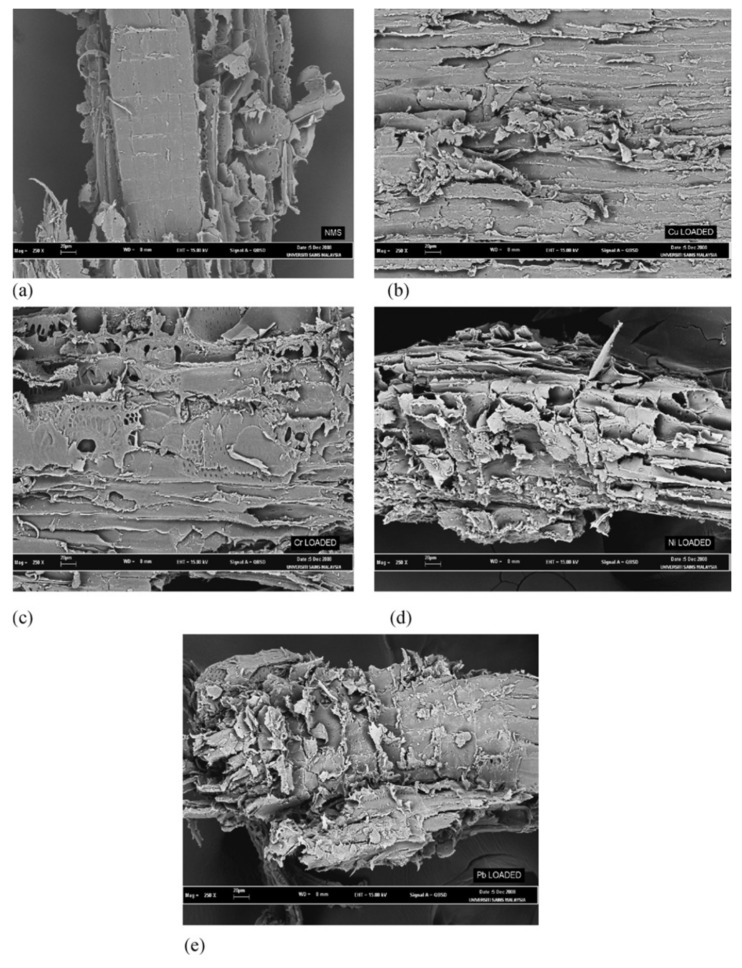
Scanning electron microscope micrograph of meranti sawdust (magnification × 250): (**a**) natural meranti sawdust, (**b**) Cu(II) loaded, (**c**) Cr(III) loaded, (**d**) Ni(II) loaded and (**e**) Pb(II) loaded. Reprinted with permission of Elsevier from Rafatullah et al., 2009.

Other methods are also applied to further determine characteristics of sawdust, such as the Brunauer–Emmet–Teller (BET) method to determine the BET surface area of a compound. For pine sawdust, the BET surface area was found to be 104.95 m^2^/g [97] and 0.62 m^2^/g for meranti sawdust [41]. This same study applied BJH (Barrett–Joiner–Halenda) to determine the average pore size of meranti sawdust, found to be 253.4 Å, which, according to International Union of Pure and Applied Chemistry (IUPAC) classification, determines the pores of the meranti sawdust structure to be mesoporous since the calculated size is more than 20 Å but less than 500 Å. Figure 3 and Figure 4 show the scanning electron microscope analysis of pine sawdust and both natural and metal-loaded meranti sawdust in order to closely examine the shape of the surface of sawdust. Both figures display a rough surface with Figure 3 showing tubular formations while Figure 4 shows some pores; all these observations lead to an increase in the surface area of the sawdust, hence increasing the sorption capacity.

### 5.2. Parameters Affecting Adsorption onto Sawdust

**Figure 5:**
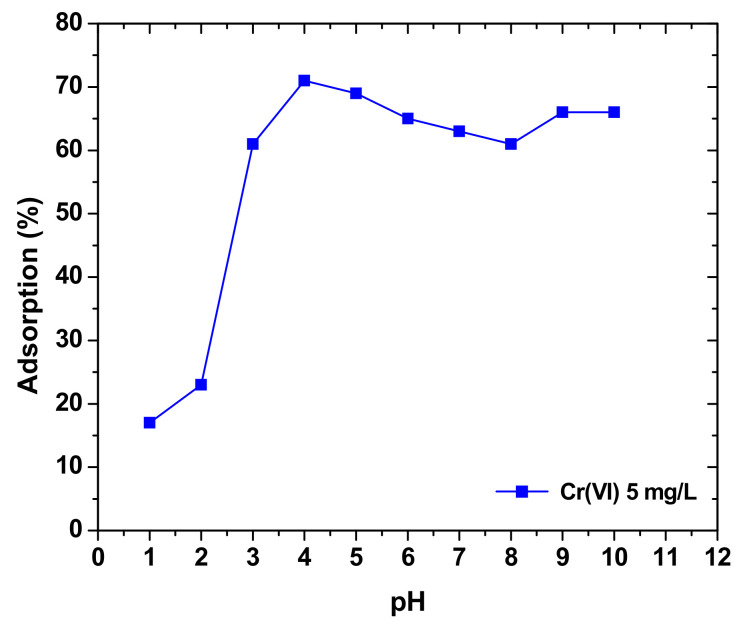
Effect of pH on the removal percentage of Cr(VI) by maple sawdust. Reprinted with permission Elsevier from Yu, L. et al., 2003.

There are many parameters that affect the sorption of heavy metals in sawdust and hence it is very essential to investigate these parameters in order to find the most suitable ways in which sawdust can achieve maximum sorption capacity and thus improve the adsorption process of heavy metals into sawdust. One of these parameters is pH. Figure 5 shows the effect of pH on removal of hexavalent chromium ions by maple sawdust. The adsorption percentage increases until a certain point when the pH of the solution is around 5 and then starts decreasing as the solution becomes more and more basic. Figure 5 shows that the adsorption percentage of Cr (VI) onto maple sawdust increases with the increase in pH until it reaches 5, which is considered the optimum pH for this sorption [99]. Another study on the effect of pH on the removal of copper ions by sawdust shows that the sawdust adsorption increases with increasing pH until the pH is 7; adsorption keeps on increasing in a pH ranging from 2 to 7 and then starts decreasing, showing that adsorption of copper ions is favored in a mainly acidic solution and keeps on increasing until it reaches the maximum adsorption which is at a pH equal to 7 [48]. *Pinus halepensis* was also subjected to pH change in order to determine its effect on adsorption of copper and lead ions into pine sawdust. The results are in accordance with the previous studies as the adsorption percentage increases with increasing pH until it reaches a maximum at a pH of 5 and a pH of 7 for lead and copper ions, respectively, and then the adsorption percentage starts to decrease with further increasing of pH [97]. It can be concluded that pH directly affects the adsorption process as adsorption of heavy metals into sawdust increases with pH until a certain level and that level varies depending on each heavy metal and this is due to the ability of pH to modify the functional groups on the surface of sawdust and change their charges.

**Figure 6:**
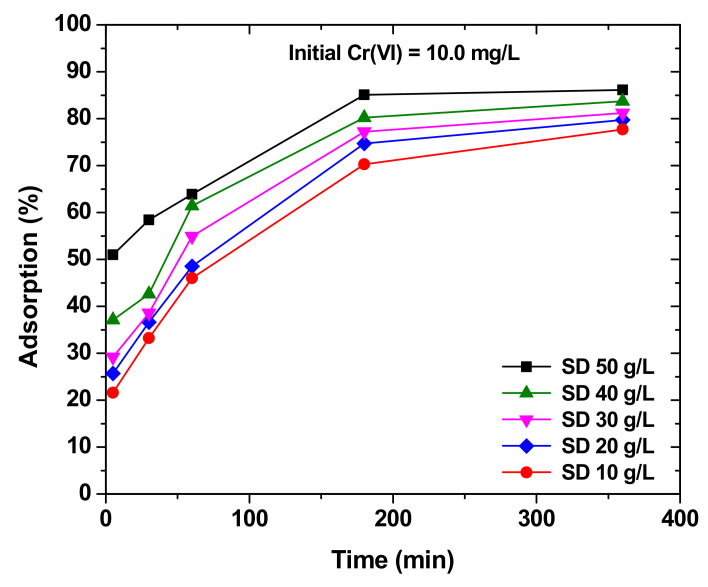
Effect of time on the removal of Cr(VI) by maple sawdust. Reprinted with permission Elsevier from Yu, L. et al., 2003.

Contact time, which is the amount of time in which heavy metals are present in the same solution as sawdust, plays an important role in the adsorption of heavy metal ions into sawdust. Figure 6 shows the adsorption of Cr(VI) into maple sawdust with respect to time. Figure 6 shows that the adsorption percentage of Cr(VI) increases with time until it becomes stable at around 200 min, which is when equilibrium is reached. It is also notable that adsorption increases rapidly in the early contact time within a few minutes, and starts to decrease gradually until equilibrium is achieved. Moreover, the sawdust concentration also affects time as it is observed that the lower the concentration of sawdust, the higher is the adsorption percentage [99]. Similar observations were found for removal of copper and lead by pine sawdust as the adsorption increased sharply in the first minutes of contact time ranging from 100% to 92% for copper in various times from 5 min to 3 depending on the concentration of copper that was put into the solution. Furthermore, lead ions experienced more or less the same results, with the only difference being a larger adsorption percentage than copper (94–100%) with contact times ranging from 5 min to 24 h [97]. It is normal that the adsorption rate is at its highest in the early contact time and this is explained by the availability of a larger surface area in order for adsorption to occur. As time passes, the surface area available for heavy metals starts to become saturated and as a result, the adsorption rate starts decreasing slowly until reaching equilibrium; after equilibrium is detected, the suitable contact time is detected and there is no point of further continuing the process.

**Figure 7:**
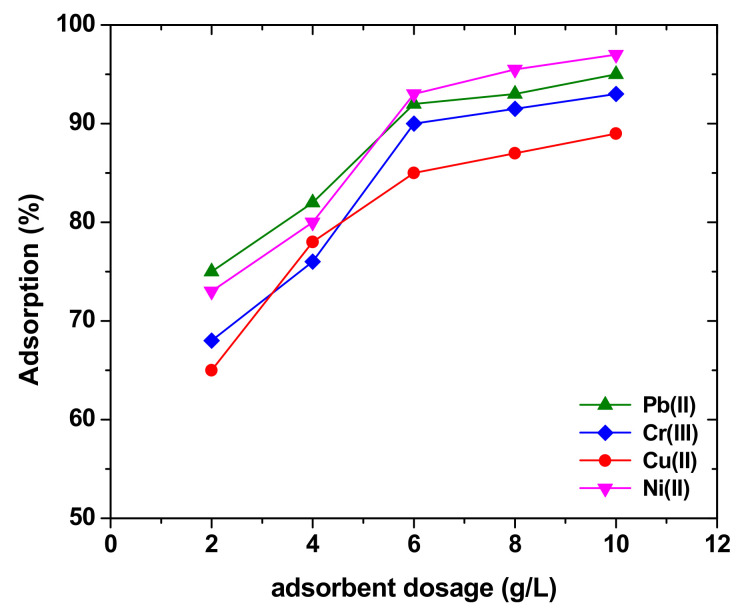
Effect of adsorbent dosage in removal of several metals by meranti sawdust. Reprinted with permission of Elsevier from Rafatullah et al., 2009.

Figure 7 shows the effect of the adsorbent dose on the adsorption process of lead, nickel, copper and trivalent chromium ions on meranti sawdust. The rate of adsorption increased from 76 to 96%, 68 to 94%, 65 to 89% and 73 to 97% for lead, trivalent chromium, copper and nickel ions, respectively [41].

The removal rate of the heavy metals is directly proportional to the concentration or dosage of the adsorbent. As the dosage of the adsorbent increases, the adsorption percentage of heavy metals into sawdust increases. This is also the case of hexavalent chromium ions as the percentage of ions binding to the maple sawdust increases with the increasing weight of maple sawdust [99]. This phenomenon can be easily explained as the higher the weight of the sawdust in the solution, the more active sites are available for the heavy metal ions to be deposited on. Interestingly, the number of metal ions present in the solution also plays a role in determining the adsorption process.

**Figure 8:**
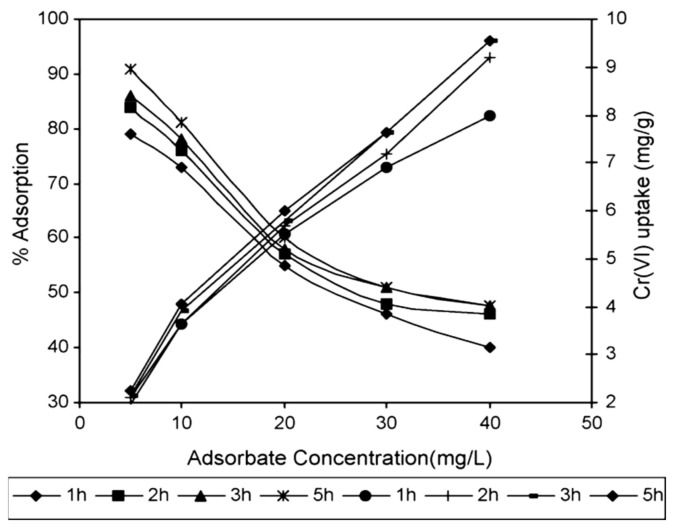
Effect of adsorbate dosage on removal of Cr(VI) by sawdust. Reprinted with permission of Elsevier from Baral et al., 2016.

Figure 8 illustrates the effect of hexavalent chromium concentrations and the metal uptake in treated sawdust. As the amount of chromium ions in the solution increases initially, the percentage of adsorption decreases but the metal uptake capacity is increased [98]. Copper ions also show the same trend as adsorption decrease as the initial concentration of copper increases when increasing the metal uptake capacity [8]. Usually, a higher initial concentration means a higher amount of heavy metal ions to be adsorbed into the active sites of sawdust and a higher ratio of ions to active sites, which increases the metal uptake capacity.

**Figure 9:**
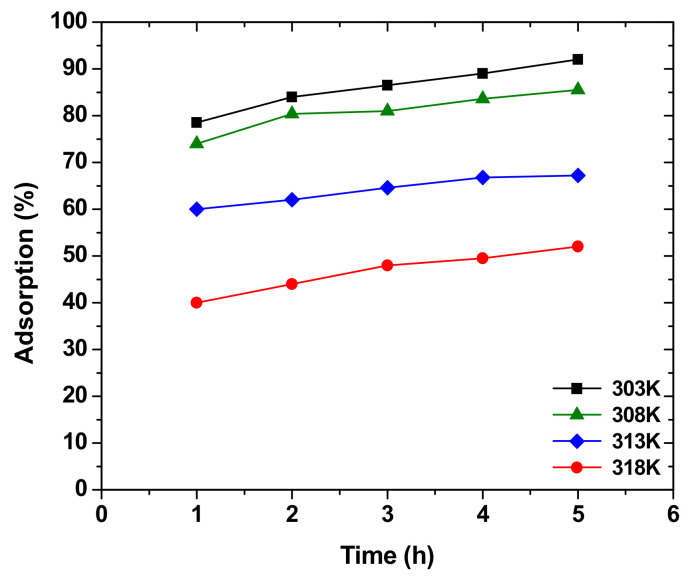
Effect of temperature on Cr(VI) adsorption by sawdust. Reprinted with permission of Elsevier from Baral et al., 2016.

Temperature plays a crucial role in the adsorption process of heavy metals and should be considered when dealing with purification of wastewater. Usually, as the temperature increases, the number of heavy metals adsorbed increases until a certain temperature [8], which is recognized as the optimum temperature. The initial increase in temperature creates more active sites due to agglomeration until reaching the optimum temperature. However, in other studies the reverse phenomenon happens. As can be shown by Figure 9, the optimum temperature of copper by sawdust was found to be 40 °C, but the increase in temperature led to a direct decrease in adsorption percentage of hexavalent chromium into treated sawdust and this is because temperature can increase the mobility of the metal ions present in the solution, including heavy metals on the adsorbent site, thus paving the way for desorption and decreasing the adsorption percentage [98].

A key point in the use of sawdust as an adsorbent material is modification in order to enhance the selectivity/capacity. As the adsorption process occurs on the surface of the sawdust, its modification can strongly affect the adsorptive capacity of this material. A number of chemical, physical, and other modification methods have been employed for this objective. In fact, to enhance the efficiency of the adsorbent, several pretreatment methods have been investigated employing various modifying agents, such as: (i) basic solutions (NaOH, Ca(OH)_2_, KOH, Na_2_CO_3_); (ii) acid solutions (HCl, H_2_SO_4_, H_3_PO_4_, CH_3_COOH [100], HNO_3_, citric acid [101]); (iii) mineral salts (NaCl, KCl, Na_2_HPO_4_, NaHCO_3_ [102]); (iv) organic compounds (ethylene diamine [103], formaldehyde, epichlorohydrin, methanol, dyes); (v) phosphorylation treatment (CO(NH_2_)_2_ (urea) + H_3_PO_4_ [102]).

A very important point for discussion is the adsorption mechanism between sawdust and heavy metal ions [104]. Most researchers [104] have assumed, with no evidence, an ion exchange mechanism that occurs between hydrogen from cellulose molecules and adsorbing metal ions. In only a few cases was it considered a possibility that Ca atoms, contained in the adsorbent molecular structure, can be exchanged with heavy metal ions [105]. Assuming a mass balance for magnesium and calcium only, supposing that these two elements predominantly act in the ion exchange process during the adsorption, it was found that the total amount of earth alkali metals transferred to the aqueous phase corresponds to the adsorption capacity of around 4 mg/g in the case of copper, thus confirming the exchange mechanism where calcium is replaced by heavy metal ions. By determining the content of alkali and alkaline earth metals in sawdust, then by measuring the quantity of the released cations (Na^+^, K^+^, Ca^2+^, Mg^2+^) during rinsing the sawdust with distilled water and by measuring the quantity of the released cations after adsorption of heavy metal ions (Cu^2+^, Zn^2+^, Ni^2+^), the adsorption mechanism can be considered as partly explained. It turns out that the residual content of sodium and potassium in sawdust changes by only 10–20%, most probably due to an additional leaching of these metals by water. Leached amounts do not exceed 10–20 μg per gram of sawdust. The remaining content of magnesium at 0.34 mg per gram of sawdust reduces to approximately 0.1 mg/g, thus indicating that this metal takes part in the adsorption process to a certain extent. The calcium content in rinsed sawdust changes significantly during the adsorption, reducing to 2.1 mg/g in the case of copper ion adsorption i.e., 2.65 and 2.85 mg/g of sawdust for nickel and zinc, respectively. Based on these considerations, it can be pointed out that predominantly calcium and to a lesser extent magnesium acts in the ion exchange process that occurs during the adsorption of heavy metal ions. Sodium and potassium are mainly washed out by water from the treated solution.

### 5.3. Adsorption Isotherms

Every adsorption process has to follow an isotherm, as isotherms determine the way in which heavy metal ions are deposited into a certain surface area. Many investigations have been conducted to investigate how heavy metal cations behave when being adsorbed by sawdust. One of the basic adsorption isotherms is the Langmuir isotherm characterized by the following equation:

(1) $$\frac{C_{e}}{q_{e}}=\frac{1}{q_{m}b}+\frac{C_{e}}{q_{m}}$$

where C_e_ is the equilibrium concentration in mg/L, q_m_ is the amount of adsorbate necessary for a monolayer to form on the mass of adsorbent in mg/g, q_e_ is the amount adsorbed on unit mass of adsorbent in mg/g and b is the adsorption equilibrium constant.

If the Langmuir isotherm is obeyed, then a plot of C_e_/q_e_ versus C_e_ should give a straight line and from this plot, q_m_ and b can be calculated by calculating the slope and the intercept.

Equation (1) can be computed into dimensionless parameters giving the following equation:

(2) $$R_{L}=\frac{1}{1+b_{c}}$$

If R_L_ has a value bigger than 1 then it represents an unfavorable adsorption. Linear adsorption is achieved when R_L_ is equal to 1, irreversible adsorption occurs when R_L_ is equal to 0 and a favorable adsorption when R_L_ lies somewhere between 0 and 1 [106].

The Freundlich isotherm is expressed by the following equations:

(3) $$q_{e}=K_{F}C_{e}{}^{n}$$

(4) $$\log\left(q_{e}\right)=\log\left(K_{F}\right)+\frac{1}{n}\log\left(C_{e}\right)$$

where K_F_ and n are the Freundlich equilibrium constants that can be determined by plotting log (q_e_) vs log (C_e_)

Table 4 shows the different parameters obtained by applying both Langmuir and Freundlich isotherms to the adsorption of copper and lead ions into pine sawdust. Both copper and lead showed a favorable adsorption since their R_L_ ranges between 0 and 1 (0.08–0.813 and 0.037–0.658 respectively).

However, the linear regression value R^2^ for both metals is considerably low (0.553 for copper and 0.492 for lead), which indicates that the following adsorption does not follow the Langmuir isotherm exactly. In fact, values obtained for linear regression of both metals for the Freundlich isotherm are better than the ones obtained in Langmuir (0.992 for copper and 0.772 for lead). This means that adsorption of copper and lead ions into sawdust follow the Freundlich isotherm, which implies a multilayer adsorption; however, since the linear regression value of lead is acceptable yet slightly low, it cannot be assured that Freundlich isotherm is the only determining step for heavy metals adsorption into pine sawdust [97]. Another study on the adsorption of cadmium and lead into pine sawdust revealed that the adsorption process followed the Langmuir model, indicating the formation of a single layer during this experiment and the incomplete fulfillment of the surface area of sawdust [69]. These two examples show that adsorption of heavy metals into sawdust are not restricted to one isotherm but can be subjected to many possible isotherms depending on many factors such as the metal and sawdust under investigation. The adsorption of heavy metals into sawdust is not unique and can be achieved either by monolayer or multilayer adsorption, and it is not the only determining step.

### 5.4. Adsorption Kinetics

Adsorption kinetics are used in order to determine the rate-determining step of adsorption of any material into the specified adsorbent. Kinetics are very important in order to design an efficient adsorption model to improve the whole process. Many adsorption models have described the adsorption process [42,106,107,108] and most of them reported that adsorption of heavy metal ions followed a pseudo second-order model [109]. A pseudo second-order model has the following equation:

(5) $$\frac{t}{q_{t}}=\frac{1}{k_{2}q_{e}{}^{2}}+t\frac{1}{q_{e}}$$

where k_2_ is the second order adsorption rate and can be determined by plotting t/q_t_ vs t.

Determining the order of the adsorption kinetics is not enough as most of the time it is not the only step, hence the intra-particle diffusion model is given as:

(6) $$q_{t}=k_{id}t^{1/2}$$

Metal adsorption into pine sawdust shows that the linear regression value of the pseudo second-order kinetic model is greater than or equal to 0.9985 determined by plotting t/q_t_ vs t from Equation (5). This suggests that lead and copper ions are directed by the pseudo second-order kinetic model when being adsorbed into pine sawdust which also means that it is a chemisorption since the kinetic model is also able to determine the type of sorption [97].

### 5.5. Adsorption Thermodynamics

There are three main thermodynamic parameters that are important for the adsorption process: enthalpy, entropy and free energy change. The equations of these parameters are listed below:

(7) $$\Delta G^{0}=-RT\ln b$$

where R is the universal gas constant (8.314 × 10^−3^ kJ mol^−1^ K^−1^), T is the temperature (K) and b is the Langmuir constant.

(8) $$\Delta G^{0}=\Delta H^{0}-T\Delta S^{0}$$

Equation (8) can be written as the following Equation (9):

(9) $$\ln b=\frac{\Delta S^{0}}{R}-\frac{\Delta H^{0}}{RT}$$

By plotting 1/T versus lnb from Equation (9), the slope and intercept can be calculated which in turn determine ΔS^0^ and ΔH^0^, then from Equation (7) the change in free energy can be obtained. For the removal of copper and lead ions by *Pinus halepensis*, the value for the change in enthalpy was found to be positive for both metals which means that the reaction was endothermic, hence requiring energy. Moreover, if the change in enthalpy is equal or less than 1 kcal/mol (3 kJ/mol), this means that the adsorption is a physisorption; if it were a chemisorption, then the change in enthalpy would have a value usually greater than 5 kcal/mol (15 kJ/mol) [110]. The values obtained for ΔH^0^ for both copper and lead are 17.5 kJ/mol and 25.5 kJ/mol, respectively, showing that the adsorption of both metals is a chemisorption. The change in free energy determines whether the reaction is spontaneous or not and since the change in free energy was positive for copper at all temperatures and for lead at low temperatures, this means that the reaction is not spontaneous under these circumstances and only adsorption of lead on pine sawdust at high temperatures can be considered as spontaneous. Since ΔS^0^ has a positive value, this shows an increase in randomness between the solid/liquid interface of the copper and lead ions and the pine sawdust [97].

## 6. Conclusions

Heavy metals pose a serious threat to the environment and its elements due to their potential environmental and health issues. There are many sources for heavy metals, mostly from processing industries. The world’s top organizations are constantly working on improving the regulations regarding the concentration of heavy metals in wastewater in order to regulate and control waste disposal. Scientific studies have focused on the adsorption processes in order to remove heavy metals from wastewaters, since previous techniques have failed or succeeded but with high operational costs. Agricultural wastes hold great interest since they are very cheap to acquire and have shown tremendous results in removing heavy metals from wastewater. Sawdust in particular has proved to be an interesting element for water purification due to its structure, which is made from cellulose, lignin and carboxyl groups which improve the ability of cations to be acquired by the active sites on sawdust. Many parameters such as pH, contact time, adsorbent and adsorbate dosage and temperature affect the adsorption of heavy metals into sawdust and must be taken into consideration to obtain the optimum conditions necessary for enhancing the adsorption process. The adsorption of heavy metals into sawdust follow the Freundlich or Langmuir isotherms, meaning that the adsorption can happen in both a monolayer or multilayer manner. The adsorption by sawdust usually follows the pseudo second-order kinetic model which means that, usually, the adsorption into sawdust is a chemisorption, but it is not always the only rate-determining step nonetheless. The thermodynamics studies in some special cases, such as in the removal of Cu(II) and Pb(II), showed that the adsorption into sawdust is an endothermic reaction and spontaneous with an increase in randomness between the solid and liquid surface of contact. It can be concluded that sawdust is a very promising material for the purification of wastewater.

## Figures and Tables

**Table 2:** The elemental compositions of various types of sawdust.

Sawdust	Elemental Compositions (%)			
	C	H	O	N
Pine sawdust	46.41	6.27	47.23	0.06
Meranti sawdust	42.38	5.27	42.41	0.14
Chinese fir sawdust	48.95	6.54	53.74	0.11
Rubber-wood sawdust	44.01	8.04	47.50	0.45
Sawdust (Canada)	45.20	6.70	48.00	0.10
Sawdust (Colombia)	48.50	6.60	44.90	0.00
Red wood sawdust	27.1	Not given	72.5	Not given
*Parkia biglobosa* sawdust	57.62	Not given	35.93	Not given

**Table 3:** Approximate composition of sawdust.

Element	Percentage (%)
Ash	1.0–1.8
Crude protein	0.9–1.2
Acid detergent fiber	26.4–34.4
Crude fiber	62.6–68.4
Dry matter	95.0–99.1

**Table 4:** Adsorption isotherm data for lead and copper into pine sawdust.

	Freundlich			Langmuir			
Metal	K_f_	n_F_	R^2^	q_m_	b	R^2^	R_L_
Cu	1.59	0.83	0.992	9.59	0.23	0.553	0.08–0.813
Pb	4.1	0.64	0.722	13.48	0.52	0.492	0.037–0.658
